# How much is enough? Hematoma clearance thresholds and 30-day outcomes after sMIS for ICH across overall and baseline volume strata

**DOI:** 10.3389/fneur.2026.1756225

**Published:** 2026-07-22

**Authors:** Lei Deng, Zhipeng Zhang, Xianbin Zhang, Jing Zha, Cunlin Gong, Yuhan Xiong, Haijun Chen, Zhenxing Yu

**Affiliations:** 1 Department of Neurosurgery, The 908th Hospital of the Joint Logistic Support of the People’s Liberation Army, Nanchang, China; 2 Department of Radiology, The 908th Hospital of the Joint Logistic Support of the People’s Liberation Army, Nanchang, China

**Keywords:** 3D slicer, hematoma clearance rate, intracerebral hemorrhage, modified Rankin scale, stereotactic minimally invasive surgery

## Abstract

**Background:**

The prognostic relevance of hematoma clearance after stereotactic minimally invasive surgery (sMIS) for supratentorial intracerebral hemorrhage (ICH) remains incompletely defined. We evaluated the association between hematoma clearance rate (HCR) and 30-day functional outcome and explored clinically interpretable HCR thresholds.

**Methods:**

We retrospectively analyzed 148 consecutive adults with spontaneous supratentorial ICH who underwent frame-based sMIS between January 2020 and December 2024. Preoperative and early postoperative non-contrast CT scans were segmented using 3D Slicer to derive preoperative hematoma volume, postoperative residual hematoma volume, intraoperative aspirated hematoma volume, and HCR. The primary outcome was 30-day unfavorable functional outcome, defined as modified Rankin Scale (mRS) 4–6. Multivariable logistic regression, generalized additive models, ROC analysis, threshold-based diagnostic performance analysis, interaction analysis, and sensitivity analysis were performed. The fully adjusted model included age, admission Glasgow Coma Scale category, preoperative hematoma volume, Barras morphology grade, international normalized ratio, hemoglobin, platelet count, onset-to-surgery time, and postoperative intracavitary thrombolysis.

**Results:**

Of 148 patients, 73 achieved mRS 0–3 and 75 had mRS 4–6 at 30 days. Higher HCR was independently associated with lower odds of unfavorable outcome in the fully adjusted model (OR 0.91 per 1% increase, 95% CI 0.86–0.96; *p* < 0.001). This association remained robust after additional adjustment for postoperative intracranial rebleeding and intracranial infection (OR 0.89, 95% CI 0.84–0.95; *p* < 0.001). HCR showed good discrimination for unfavorable outcome, with an AUC of 0.837 and an approximate threshold near 60%. Compared with postoperative residual hematoma volume >15 mL, HCR < 60% showed higher sensitivity, negative predictive value, accuracy, and threshold-based AUC, whereas residual hematoma volume >15 mL showed higher specificity and positive predictive value.

**Conclusion:**

HCR after sMIS was independently associated with 30-day functional outcome and may serve as a postoperative prognostic benchmark. An HCR threshold near 60% may help risk stratification after sMIS and may complement absolute residual-volume thresholds such as 15 mL. Prospective multicenter validation is required before this threshold can be used as a prescriptive treatment target.

## Introduction

Intracerebral hemorrhage (ICH) remains one of the most devastating forms of stroke, accounting for approximately 10–15% of all strokes worldwide and associated with high rates of mortality and long-term disability. Despite improvements in neurocritical care, short-term mortality remains substantial, and many survivors experience persistent functional dependence (1). The absence of broadly effective targeted therapies has led to increasing interest in minimally invasive surgical evacuation of hematoma to relieve mass effect and reduce secondary injury related to blood-derived cytotoxic and inflammatory mediators (2, 3). Stereotactic minimally invasive surgery (sMIS), including stereotactic puncture, aspiration, catheter drainage, and selective intracavitary thrombolysis, has been widely adopted because it may reduce tissue disruption compared with conventional open craniotomy (4). However, clinical outcomes after minimally invasive evacuation remain heterogeneous, partly because benefit may depend not only on patient selection and hematoma location, but also on the extent and quality of hematoma removal (5). Recent evidence suggests that the degree of hematoma evacuation, rather than surgical intervention alone, plays a pivotal role in determining functional recovery. Early and effective removal of hematoma has been associated with lower remnant cavity burden and improved neurological outcomes (6).

Several studies have highlighted hematoma clearance rate (HCR) and postoperative residual hematoma volume as quantitative indicators of surgical efficacy. For example, prior catheter-based series have suggested that patients achieving higher evacuation rates, such as ≥70%, may have better functional recovery, whereas analyses related to MISTIE III emphasized the clinical relevance of achieving a small residual hematoma volume, commonly represented by a residual-volume threshold of ≤15 mL (7). Similarly, early and adequate removal of ICH has been shown to correlate with improved long-term function, reduced postoperative cavity volume, and lower mortality (8). Yet, the optimal clearance threshold and its prognostic value remain poorly defined, and whether the predictive capacity of HCR differs across hematoma volume subgroups has not been addressed. Furthermore, patient selection is critical for maximizing benefit from minimally invasive techniques. Subgroup analyses indicate that specific populations, such as supratentorial deep hematomas, may derive more substantial benefit compared with lobar or infratentorial hemorrhages (9). Prior studies also suggested that morphological characteristics (e.g., Barras grade irregularity) may influence surgical feasibility and outcomes (10). Nevertheless, the optimal way to quantify and interpret surgical clearance remains uncertain. Percentage clearance and absolute residual volume may capture related but distinct aspects of evacuation: residual volume reflects the remaining postoperative clot burden, whereas HCR incorporates baseline hematoma size and therefore reflects relative evacuation efficiency.

Several clinically important factors may also influence both HCR and short-term outcomes after sMIS. Baseline hematoma volume, admission neurological severity, coagulation status, and hematoma morphology may affect both the feasibility of evacuation and the risk of unfavorable outcome. In particular, irregular hematoma morphology, such as Barras grade III–V, may reflect greater procedural complexity and a higher-risk hematoma phenotype. In addition, time from symptom onset to surgery, postoperative intracavitary thrombolysis, and major postoperative complications such as rebleeding or intracranial infection may influence the relationship between surgical clearance and functional recovery. Therefore, evaluating HCR without accounting for these factors may overestimate or misinterpret its prognostic value.

Accordingly, we conducted a single-center retrospective cohort study of adults with spontaneous supratentorial ICH treated with sMIS. We aimed to evaluate the association between HCR and 30-day functional outcome, explore clinically interpretable HCR thresholds, compare the threshold-based performance of HCR with the established postoperative residual-volume threshold of 15 mL, and examine whether the HCR–outcome association varied across baseline hematoma-volume strata or Barras morphology groups. Because this was an observational study, HCR was interpreted as an associative postoperative prognostic marker rather than as evidence of a causal treatment target.

## Methods

### Ethics and reporting

The study adhered to the Declaration of Helsinki and was approved by the Ethics Committee of The 908th Hospital of the Joint Logistic Support Force of the Chinese People’s Liberation Army. Given the retrospective design and use of de-identified clinical and imaging data, the requirement for written informed consent was waived by the ethics committee. Reporting followed the Strengthening the Reporting of Observational Studies in Epidemiology (STROBE) recommendations.

### Study design and participants

We conducted a single-center retrospective cohort study of consecutive adults with spontaneous supratentorial intracerebral hemorrhage (ICH) treated with stereotactic minimally invasive surgery (sMIS). Consecutive patients treated at The 908th Hospital of the Joint Logistic Support Force of the Chinese People’s Liberation Army between January 2020 and December 2024 were identified through the electronic medical record system and imaging archive. Spontaneous ICH was confirmed on admission non-contrast head CT, and secondary causes were excluded using clinical records and CT angiography when available.

The inclusion criteria were as follows: (1) age 18–90 years; (2) spontaneous supratentorial ICH confirmed by non-contrast head CT or CT angiography; (3) hypertensive supratentorial ICH located in the basal ganglia, thalamus, or lobar regions, with or without intraventricular extension, but not isolated intraventricular, brainstem, or cerebellar hemorrhage; (4) treatment with stereotactic minimally invasive puncture, aspiration, and catheter drainage within the institutional surgical time window after symptom onset; and (5) availability of both preoperative and postoperative CT images for hematoma volumetry.

The exclusion criteria were as follows: (1) secondary ICH due to head trauma, ruptured aneurysm or arteriovenous malformation, brain tumor, hemorrhagic transformation of ischemic stroke, venous sinus thrombosis, or other identified causes; (2) brainstem, cerebellar, or isolated intraventricular hemorrhage; (3) previous craniotomy for hematoma evacuation before admission or conversion to open craniotomy during the index hospitalization; (4) severe systemic comorbidities expected to markedly influence short-term prognosis, such as advanced heart failure, respiratory failure, hepatic or renal failure, active malignancy, or ongoing gastrointestinal bleeding; (5) known coagulation disorders or current use of anticoagulants or strong antiplatelet agents that could not be discontinued or reversed promptly; (6) pregnancy or lactation; and (7) missing key clinical or imaging data or inability to complete 30-day follow-up.

### Participants

**Figure 1:**
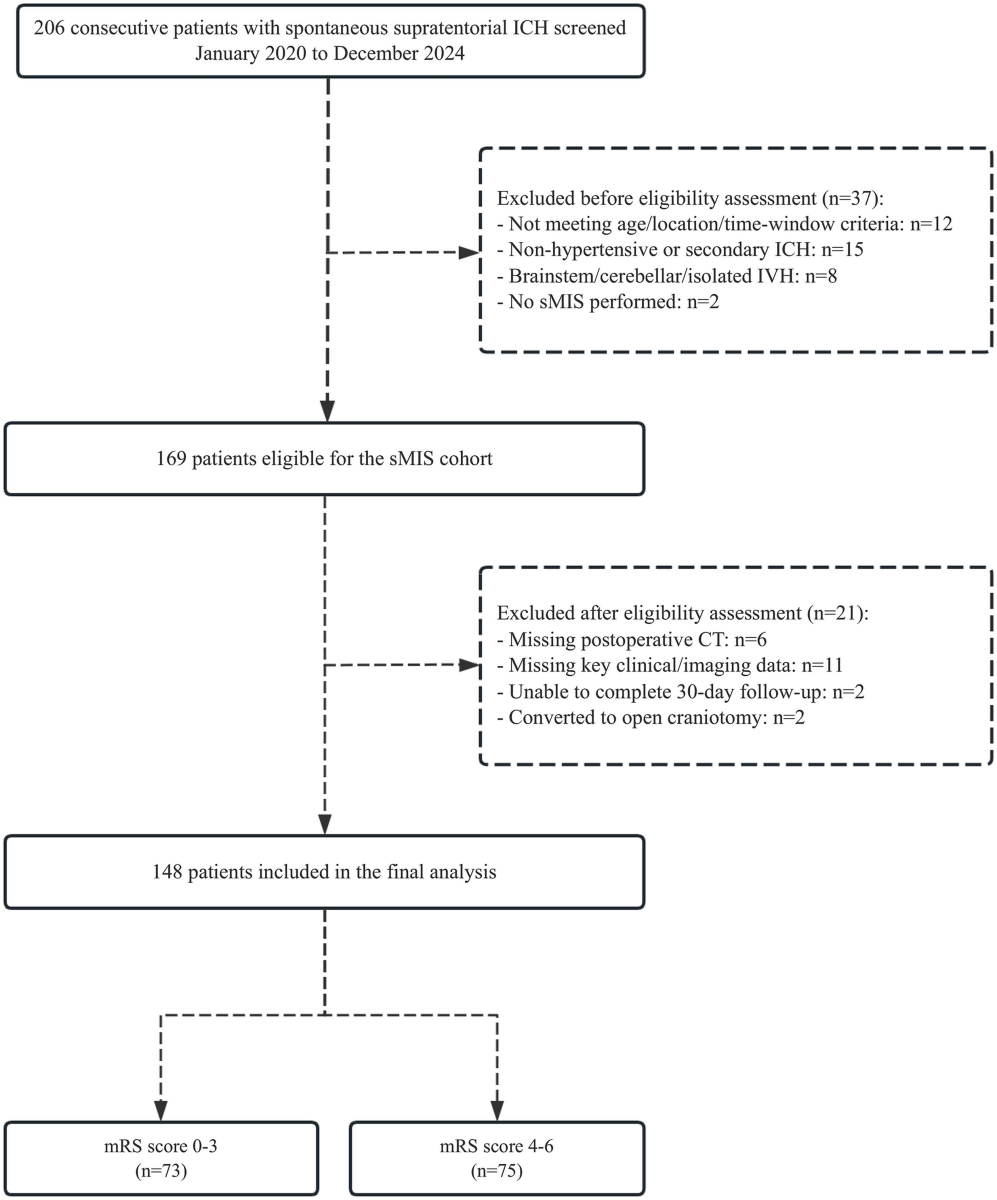
Flow diagram of patient selection. Among 206 consecutive patients with spontaneous supratentorial intracerebral hemorrhage screened between January 2020 and December 2024, 148 patients who underwent stereotactic minimally invasive surgery and had complete clinical, imaging, and 30-day follow-up data were included in the final analysis. The specific reasons for exclusion at each step are shown in the diagram. ICH, intracerebral hemorrhage; IVH, intraventricular hemorrhage; mRS, modified Rankin Scale; sMIS, stereotactic minimally invasive surgery.

A total of 206 consecutive patients with spontaneous supratentorial ICH were screened. Thirty-seven patients were excluded before eligibility assessment because of not meeting age, location, or time-window criteria, non-hypertensive or secondary ICH, brainstem/cerebellar/isolated intraventricular hemorrhage, or no sMIS performed. Among 169 eligible patients, 21 were excluded after eligibility assessment because of missing postoperative CT, missing key clinical or imaging data, inability to complete 30-day follow-up, or conversion to open craniotomy. Finally, 148 patients were included in the analysis (Figure 1).

## Imaging acquisition and measurements

### CT acquisition

All patients underwent non-contrast head CT (NCCT) on admission (baseline CT) and a follow-up CT after stereotactic minimally invasive surgery (postoperative CT). Baseline CT was performed within 1 h of hospital arrival; sMIS was completed within 24 h of admission; the first postoperative CT was obtained within 24–48 h after surgery. Axial images were reconstructed at ≤5 mm slice thickness with no interslice gap according to routine brain protocol. CT angiography was additionally reviewed when available to exclude secondary causes. Two raters (a neurosurgeon and a neuroradiologist with >5 years’ experience), blinded to clinical data and outcomes, performed all measurements independently; discrepancies were resolved by consensus with a third senior reviewer. DICOM series were exported and analyzed in 3D Slicer (version 5.7.0; www.slicer.org) (11, 12). Hematoma volume was segmented on baseline non-contrast CT using 3D Slicer. We applied a semi-automatic workflow (thresholding/region-growing) with slice-by-slice manual refinement, followed by 3D label-map generation to compute volumes (mL) and surface renderings. This approach follows prior work using 3D Slicer to quantify ICH burden on CT.

### Volume computation

Hematoma volume (mL) was calculated automatically by 3D Slicer as:

$$V=N_{\text{voxels}}\times (\text{voxel}\;{\text{size}}_{x}\times {\text{voxel size}}_{y}\times \text{slice thickness}).$$

Preoperative hematoma volume was denoted V_pre_ and postoperative residual volume V_post_. The hematoma clearance rate (HCR) was defined as:

$$\mathrm{HCR}(\%)=\frac{V_{\mathrm{pre}}-V_{\text{post}}}{V_{\mathrm{pre}}}\times 100$$

### Barras morphology grading and measurement reliability

**Figure 2:**
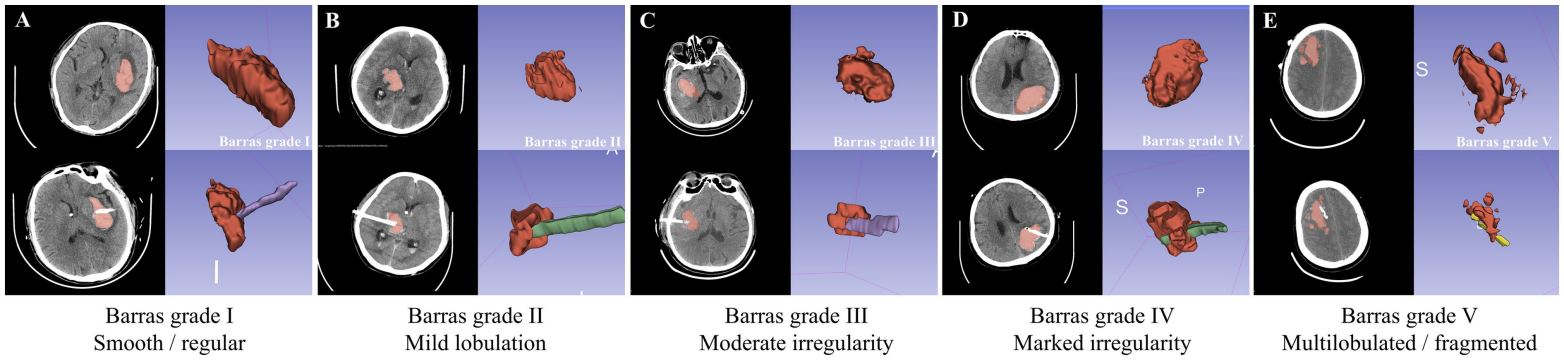
Representative Barras morphology grades on axial CT and 3D reconstructions. **(A–E)** show Barras grades I–V, respectively: grade I, smooth/regular; grade II, mild lobulation; grade III, moderate irregularity; grade IV, marked irregularity; and grade V, multilobulated or fragmented morphology. The left subpanels show axial non-contrast CT with segmented hematoma, and the right subpanels show corresponding reconstructions generated using 3D Slicer. Where visible, the catheter trajectory is indicated on postoperative images.

Hematoma morphology was graded on baseline CT using the Barras scale (grades I–V) (Figure 2) (I-V) (10). Grades I–II were considered regular morphology, and grades III–V were considered irregular morphology. Grading was performed on axial CT images with support from three-dimensional surface reconstructions to distinguish smooth, lobulated, irregular, or fragmented hematoma contours. Inter-rater reliability was assessed for Barras morphology grading and hematoma volumetry. Barras grades were independently assigned by two raters blinded to clinical outcome. Agreement for the ordinal Barras grades I–V was evaluated using weighted Cohen’s *κ*, and agreement for the dichotomized Barras morphology group (I–II vs. III–V) was evaluated using Cohen’s κ. Inter-rater reliability for preoperative and postoperative hematoma volume measurements was assessed using intraclass correlation coefficients (ICCs) based on a two-way random-effects model for absolute agreement.

### Surgical technique and perioperative management

All procedures were performed using a frame-based stereotactic system. After stereotactic frame fixation, CT images were obtained and imported into the stereotactic planning system (13, 14). After induction of general anesthesia, the stereotactic frame was secured to the skull with care to patient comfort and stability. A non-contrast CT was then obtained with the frame in place and imported into the stereotactic planning software. Using these images, the surgeon defined the hematoma target and calculated Cartesian coordinates and a safe trajectory/entry angle that avoided eloquent cortex, deep perforators, and ventricles whenever possible. In the operating room, the head was prepared and draped. A small linear incision was made at the planned entry site, and a burr hole was fashioned using a high-speed drill with a side-cutting bit. Guided by the preplanned coordinates, a stereotactic biopsy cannula was advanced through the burr hole to the target. An aspiration catheter was then introduced through the cannula and the clot was evacuated in a controlled fashion with gentle negative pressure, keeping the catheter tip within the hematoma cavity and avoiding wall suctioning. Hemostasis was confirmed; the catheter/cannula was withdrawn, and the wound was closed in layers. Immediately after surgery, a CT scan was obtained to document hematoma reduction and to screen for complications such as rebleeding or malposition (Figure 2). Standardized neurocritical care (blood pressure control, reversal of coagulopathy when indicated, intracranial pressure management) was provided per guideline-based pathways (15).

All patients received standardized neurocritical care according to institutional protocols, including blood pressure control, reversal of coagulopathy when indicated, intracranial pressure management when required, head-of-bed elevation, maintenance of normoglycemia and normothermia, and prophylaxis against deep-vein thrombosis. Any neurological deterioration triggered urgent imaging and targeted management. Postoperative intracavitary thrombolysis was defined as urokinase administration through the drainage catheter after postoperative imaging confirmed catheter position and excluded active rebleeding. Thrombolysis status was recorded as a binary variable. Postoperative intracranial rebleeding was defined as new hemorrhage or hematoma enlargement on follow-up CT after sMIS. Postoperative intracranial infection was defined according to clinical documentation, cerebrospinal fluid findings when available, imaging evidence, or requirement for targeted antimicrobial treatment. Other postoperative complications, including lung infection, hydrocephalus, epilepsy, and second surgery, were extracted from medical records. The decision to administer urokinase, the number of administrations, and treatment discontinuation were determined by the treating neurosurgical team according to postoperative CT findings, catheter position, residual clot burden, and bleeding risk.

### Outcome assessment and blinding

The primary outcome was 30-day functional status, assessed using the modified Rankin Scale (mRS) and dichotomized as favorable outcome (mRS 0–3) and unfavorable outcome (mRS 4–6). During data verification for this revision, 30-day mRS outcomes were independently re-reviewed by two trained investigators who were not involved in the surgical procedures and were blinded to imaging-derived hematoma volumes, HCR values, and Barras morphology grading. Imaging raters and outcome assessors were independent of each other (16) (Table 1). The secondary outcome was 30-day all-cause mortality.

**Table 1:** The modified Rankin scale (mRS).

Level	Details of the modified rankin scale
0	No symptoms.
1	No significant disability. Able to carry out all usual activities, despite some symptoms.
2	Slight disability. Able to look after own affairs without assistance, but unable to carry out all previous activities.
3	Moderate disability. Requires some help, but able to walk unassisted.
4	Moderately severe disability. Unable to attend to own bodily needs without assistance, and unable to walk unassisted.
5	Severe disability. Requires constant nursing care and attention, bedridden, incontinent.
6	Dead.

### Covariates

The following covariates were extracted from electronic medical records and imaging data: age, sex, smoking history, alcohol use, hypertension, previous cerebral hemorrhage, previous cerebral infarction, admission systolic and diastolic blood pressure, admission GCS score, preoperative hematoma volume, postoperative residual hematoma volume, intraoperative aspirated hematoma volume, HCR, Barras morphology grade, number of puncture catheters, onset-to-surgery time, postoperative intracavitary thrombolysis, postoperative intracranial rebleeding, postoperative intracranial infection, postoperative lung infection, hydrocephalus, epilepsy, second surgery, and laboratory indices including white blood cell count, red blood cell count, hemoglobin, platelet count, prothrombin time, activated partial thromboplastin time, fibrinogen, thrombin time, and international normalized ratio.

### Statistical analysis

Continuous variables were summarized as mean ± SD or median (interquartile range [IQR]), as appropriate, and categorical variables were summarized as counts and percentages. Patients were grouped according to 30-day functional outcome (mRS 0–3 vs. mRS 4–6). Continuous variables were compared using the t-test or Mann–Whitney U test, as appropriate, and categorical variables were compared using the *χ*^2^ test or Fisher’s exact test when expected cell counts were small. The primary exposures were intraoperative aspirated hematoma volume and HCR. Univariable logistic regression was performed to identify factors associated with 30-day unfavorable functional outcome. Multivariable logistic regression was then used to evaluate the associations of intraoperative aspirated hematoma volume and HCR with 30-day unfavorable outcome. Model 1 adjusted for age, admission GCS category, preoperative hematoma volume, Barras morphology grade, and INR. Model 2 adjusted for Model 1 covariates plus hemoglobin, platelet count, onset-to-surgery time, and postoperative intracavitary thrombolysis. Both continuous and quartile-based exposure models were fitted, and linear trends across quartiles were evaluated by modeling quartile categories as an ordinal variable.

Generalized additive logistic models were used to flexibly depict the associations of HCR and intraoperative aspirated hematoma volume with 30-day outcome, overall and across preoperative hematoma volume strata. Preoperative hematoma volume strata were defined as ≤20 mL, >20–40 mL, and >40 mL. Receiver-operating characteristic (ROC) analyses were used to evaluate discrimination, and optimal cut-offs were determined using the Youden index. To compare clinically interpretable thresholds, threshold-based diagnostic performance was calculated for HCR < 60% and postoperative residual hematoma volume >15 mL for predicting 30-day unfavorable outcome, including sensitivity, specificity, positive predictive value, negative predictive value, accuracy, positive likelihood ratio, negative likelihood ratio, diagnostic odds ratio, and threshold-based AUC. Multicollinearity among variables included in the fully adjusted multivariable models was assessed using variance inflation factors (VIFs). Admission GCS category was dummy-coded with GCS 3–8 as the reference group, and Barras morphology grade was coded as III–V versus I–II. A VIF value <5 was considered to indicate no substantial multicollinearity.

Effect modification was assessed by adding multiplicative interaction terms to the fully adjusted logistic regression models. Specifically, interactions between HCR and preoperative hematoma volume strata, and between HCR and Barras morphology grade, were evaluated. HCR was modeled as a continuous variable per 1% increase in the interaction analyses. Because postoperative complications may occur after exposure assessment and may partly lie on the pathway between surgical course and functional outcome, they were not included in the primary multivariable model. To evaluate whether major postoperative complications influenced the HCR–outcome association, sensitivity analysis was performed by additionally adjusting the fully adjusted HCR model for postoperative intracranial rebleeding and postoperative intracranial infection. To assess potential selection bias, key baseline characteristics were compared between patients included in the final analysis and patients excluded after eligibility assessment. All analyses were performed using R (version 4.5.1) and EmpowerStats (X&Y solutions, Boston, MA, USA). A two-sided *p* value <0.05 was considered statistically significant.

## Results

### Patient selection and 30-day outcomes

A total of 206 consecutive patients with spontaneous supratentorial ICH were screened between January 2020 and December 2024. Thirty-seven patients were excluded before eligibility assessment, including 12 who did not meet age, location, or time-window criteria, 15 with non-hypertensive or secondary ICH, 8 with brainstem, cerebellar, or isolated intraventricular hemorrhage, and 2 who did not undergo sMIS. Among 169 eligible patients, 21 were further excluded because of missing postoperative CT (*n* = 6), missing key clinical or imaging data (*n* = 11), inability to complete 30-day follow-up (*n* = 2), or conversion to open craniotomy (*n* = 2). Finally, 148 patients were included in the final analysis, including 73 patients with 30-day mRS 0–3 and 75 patients with mRS 4–6 (Figure 1). Eight patients died within 30 days, corresponding to a 30-day mortality rate of 5.41% (see Tables 2, 3). To evaluate potential selection bias, key baseline characteristics were compared between the 148 included patients and the 21 patients excluded after eligibility assessment (Table 4). No statistically significant differences were observed in age (62.77 ± 11.48 vs. 63.5 ± 13.2 years; *p* = 0.812), sex (male: 71.6% vs. 66.7%; *p* = 0.640), preoperative hematoma volume (38.95 ± 24.58 vs. 46.8 ± 32.4 mL; *p* = 0.296), admission SBP (156.4 ± 34.0 vs. 163.0 ± 35.5 mmHg; *p* = 0.432), admission DBP (89.3 ± 18.5 vs. 92.0 ± 19.1 mmHg; *p* = 0.544), or preoperative INR (1.15 ± 0.31 vs. 1.19 ± 0.36; *p* = 0.636) between included and excluded patients.

**Table 2:** The 30-day mortality in patients with spontaneous supratentorial hypertensive intracerebral hemorrhage.

Outcome	Number of cases (*n*)	Deaths (n)	Survivors (n)	Mortality rate (%)
30-day mortality	148	8	140	5.41

**Table 3:** The 30-day mRS score in patients with spontaneous supratentorial hypertensive intracerebral hemorrhage.

30-day mRS score	Number of cases (*n*)	Group	Proportion of cases (%)
Favorable outcome	*n* = 73	0–3	49.32
Unfavorable outcome	*n* = 75	4–6	50.68

**Table 4:** Baseline characteristics of participants (*n* = 148).

30-day mRS score	Favorable outcome	Unfavorable outcome	*p*-value
	0–3 points	4–6 points	
Demographic and clinical characteristics			
N	73	75	
Age, years	58.04 ± 11.42	67.37 ± 9.56	<0.001
Sex, *n* (%)			0.175
Female	17 (23.29%)	25 (33.33%)	
Male	56 (76.71%)	50 (66.67%)	
Medical history			
Smoking, *n* (%)			0.776
No	54 (73.97%)	57 (76.00%)	
Yes	19 (26.03%)	18 (24.00%)	
Alcohol use, *n* (%)			0.618
No	58 (79.45%)	62 (82.67%)	
Yes	15 (20.55%)	13 (17.33%)	
Hypertension, *n* (%)			0.611
No	14 (19.18%)	12 (16.00%)	
Yes	59 (80.82%)	63 (84.00%)	
Previous cerebral hemorrhage, *n* (%)			0.032
No	68 (93.15%)	61 (81.33%)	
Yes	5 (6.85%)	14 (18.67%)	
Previous cerebral infarction, *n* (%)			0.012
No	66 (90.41%)	56 (74.67%)	
Yes	7 (9.59%)	19 (25.33%)	
Admission status and imaging characteristics			
GCS score, *n* (%)			<0.001
3–8 points	23 (31.51%)	58 (77.33%)	
9–12 points	47 (64.38%)	16 (21.33%)	
13–15 points	3 (4.11%)	1 (1.33%)	
Admission SBP, mmHg	156.47 ± 30.90	156.37 ± 36.99	0.730
Admission DBP, mmHg	88.07 ± 16.84	90.44 ± 20.08	0.568
Preoperative HV, mL	28.20 ± 12.62	49.40 ± 28.64	<0.001
Postoperative HV, mL	10.01 ± 6.04	24.70 ± 15.25	<0.001
HV clearance rate (%)	65.03 ± 10.32	50.06 ± 10.69	<0.001
Barras morphology grade			<0.001
I–II	52 (71.23%)	10 (13.33%)	
III–V	21 (28.77%)	65 (86.67%)	
Laboratory parameters			
Pre WBC, ×10^9^/L	11.18 ± 3.36	10.68 ± 3.94	0.408
Pre RBC, ×10^12^/L	4.35 ± 0.62	4.13 ± 0.70	0.047
Pre HGB, g/L	130.44 ± 18.73	121.09 ± 23.41	0.014
Pre PLT, ×10^9^/L	204.15 ± 63.16	159.88 ± 64.42	<0.001
Pre PT, s	12.24 ± 1.34	13.85 ± 4.02	0.001
Pre APTT, s	24.76 ± 4.88	28.73 ± 11.62	0.008
Pre FIB, g/L	2.64 ± 0.74	2.66 ± 0.91	0.752
Pre TT, s	18.26 ± 1.98	19.47 ± 3.13	0.006
Pre INR	1.07 ± 0.12	1.23 ± 0.40	<0.001
Perioperative characteristics			
Onset-to-surgery time, hours	7.00 ± 3.33	6.70 ± 3.48	0.585
Number of puncture catheters, *n* (%)			0.001
1	65 (89.04%)	50 (66.67%)	
2	8 (10.96%)	25 (33.33%)	
Intraoperative aspiration of HV, mL	18.20 ± 8.06	24.70 ± 15.61	0.002
Postoperative thrombolysis, *n* (%)			0.261
No	2 (2.74%)	5 (6.67%)	
Yes	71 (97.26%)	70 (93.33%)	
Postoperative complications and mortality			
Postoperative intracranial rebleeding, *n* (%)			<0.001
No	71 (97.26%)	59 (78.67%)	
Yes	2 (2.74%)	16 (21.33%)	
Postoperative intracranial infection, *n* (%)			0.424
No	71 (97.26%)	71 (94.67%)	
Yes	2 (2.74%)	4 (5.33%)	
Postoperative lung infection, *n* (%)			0.484
No	6 (8.22%)	4 (5.33%)	
Yes	67 (91.78%)	71 (94.67%)	
Postoperative hydrocephalus, *n* (%)			0.324
No	72 (98.63%)	72 (96.00%)	
Yes	1 (1.37%)	3 (4.00%)	
Postoperative epilepsy, *n* (%)			0.726
No	70 (95.89%)	71 (94.67%)	
Yes	3 (4.11%)	4 (5.33%)	
Second surgery, *n* (%)			0.157
No	71 (97.26%)	69 (92.00%)	
Yes	2 (2.74%)	6 (8.00%)	
30-day Mortality, *n* (%)			0.004
Survival	73 (100.00%)	67 (89.33%)	
Death	0 (0.00%)	8 (10.67%)	

GCS, Glasgow Coma Scale; SBP, systolic blood pressure; DBP, diastolic blood pressure; HV, hematoma volume; WBC, white blood cell; RBC, Red blood cell; HGB, hemoglobin; PLT, platelet; PT, prothrombin time; APTT, activated partial thromboplastin time; FIB, fibrinogen; TT, thrombin time; INR, international normalized ratio; Pre, Preoperative.

### Baseline, perioperative, and postoperative characteristics

The baseline, perioperative, laboratory, imaging, and postoperative characteristics of the included patients are summarized in Table 5. Compared with patients with favorable outcome, patients with unfavorable outcome were older (67.37 ± 9.56 vs. 58.04 ± 11.42 years; *p* < 0.001), had lower admission GCS scores (GCS 3–8: 77.33% vs. 31.51%; *p* < 0.001), larger preoperative hematoma volumes (49.40 ± 28.64 vs. 28.20 ± 12.62 mL; *p* < 0.001), larger postoperative residual hematoma volumes (24.70 ± 15.25 vs. 10.01 ± 6.04 mL; *p* < 0.001), and lower HCR values (50.06 ± 10.69% vs. 65.03 ± 10.32%; *p* < 0.001). Irregular Barras morphology was also more frequent in the unfavorable outcome group than in the favorable outcome group (Barras III–V: 86.67% vs. 28.77%; *p* < 0.001). Laboratory parameters also differed between groups. Patients with unfavorable outcome had lower hemoglobin (121.09 ± 23.41 vs. 130.44 ± 18.73 g/L; *p* = 0.014), lower platelet count (159.88 ± 64.42 vs. 204.15 ± 63.16 × 10^9^/L; *p* < 0.001), and higher INR (1.23 ± 0.40 vs. 1.07 ± 0.12; *p* < 0.001). Onset-to-surgery time did not differ significantly between the favorable and unfavorable groups (6.70 ± 3.48 vs. 7.00 ± 3.33 h; *p* = 0.585). Postoperative intracavitary thrombolysis was used in most patients and was not significantly different between groups (93.33% vs. 97.26%; *p* = 0.261). Postoperative intracranial rebleeding was more frequent in the unfavorable outcome group (21.33% vs. 2.74%; *p* < 0.001), whereas postoperative intracranial infection, lung infection, hydrocephalus, epilepsy, and second surgery did not differ significantly between groups.

**Table 5:** Univariable logistic regression analysis for factors associated with 30-day unfavorable functional outcome.

Covariate	OR (95%CI)	*p*-value
Sex		
Female	Ref	
Male	0.61 (0.29, 1.25)	0.177
Age	1.09 (1.05, 1.13)	<0.001
Medical history		
Smoking		
No	Ref	
Yes	0.90 (0.43, 1.89)	0.776
Alcohol use		
No	Ref	
Yes	0.81 (0.36, 1.85)	0.618
Hypertension		
No	Ref	
Yes	1.25 (0.53, 2.91)	0.612
Previous cerebral hemorrhage		
No	Ref	
Yes	3.12 (1.06, 9.17)	0.038
Previous cerebral infarction		
No	Ref	
Yes	3.20 (1.25, 8.16)	0.015
GCS score		
3–8 points	Ref	
9–12 points	0.13 (0.06, 0.28)	<0.001
13–15 points	0.13 (0.01, 1.34)	0.087
Preoperative HV, mL	1.05 (1.03, 1.07)	<0.001
Postoperative HV, mL	1.16 (1.10, 1.22)	<0.001
Intraoperative aspiration of HV, mL	1.05 (1.02, 1.08)	0.003
HV clearance rate, per 1% increase	0.88 (0.85, 0.92)	<0.001
Number of puncture catheters		
1	Ref	
2	4.06 (1.69, 9.77)	0.0012
Barras morphology grade		
I–II	Ref	
III–V	16.10 (6.97, 37.16)	<0.001
SBP	1.00 (0.99, 1.01)	0.987
DBP	1.01 (0.99, 1.03)	0.437
Pre WBC	0.96 (0.88, 1.05)	0.406
Pre RBC	0.60 (0.36, 1.00)	0.050
Pre HGB	0.98 (0.96, 1.00)	0.010
Pre PLT	0.99 (0.98, 0.99)	0.002
Pre PT	1.35 (1.09, 1.66)	0.005
Pre APTT	1.11 (1.03, 1.19)	0.005
Pre FIB	1.03 (0.70, 1.52)	0.880
Pre TT	1.21 (1.05, 1.39)	0.009
Pre INR	25.95 (2.85, 235.93)	0.004
Onset-to-surgery time, hours	0.97 (0.89, 1.07)	0.583
Postoperative thrombolysis		
No	Ref	
Yes	0.39 (0.07, 2.10)	0.276
Postoperative intracranial rebleeding		
No	Ref	
Yes	9.63 (2.13, 43.58)	0.003
Postoperative intracranial infection		
No	Ref	
Yes	2.00 (0.35, 11.27)	0.432
Postoperative lung infection		
No	Ref	
Yes	1.59 (0.43, 5.88)	0.487
Postoperative hydrocephalus		
No	Ref	
Yes	3.00 (0.30, 29.53)	0.346
Postoperative epilepsy		
No	Ref	
Yes	1.31 (0.28, 6.09)	0.727
Second surgery		
No	Ref	
Yes	3.09 (0.60, 15.82)	0.176

GCS, Glasgow Coma Scale; SBP, systolic blood pressure; DBP, diastolic blood pressure; HV, hematoma volume; WBC, white blood cell count; RBC, red blood cell count; HGB, hemoglobin; PLT, platelet count; PT, prothrombin time; APTT, activated partial thromboplastin time; FIB, fibrinogen; TT, thrombin time; INR, international normalized ratio; CI, confidence interval; OR, odds ratio. The outcome was unfavorable 30-day functional outcome, defined as mRS 4–6.

### Univariable logistic regression analysis

Univariable logistic regression results are shown in Table 6. Older age was associated with higher odds of 30-day unfavorable functional outcome (OR 1.09, 95% CI 1.05–1.13; *p* < 0.001). Compared with admission GCS 3–8, admission GCS 9–12 was associated with lower odds of unfavorable outcome (OR 0.13, 95% CI 0.06–0.28; *p* < 0.001), whereas admission GCS 13–15 showed a similar direction but did not reach statistical significance (OR 0.13, 95% CI 0.01–1.34; *p* = 0.087). Larger preoperative hematoma volume (OR 1.05 per 1 mL increase, 95% CI 1.03–1.07; *p* < 0.001), larger postoperative residual hematoma volume (OR 1.16 per 1 mL increase, 95% CI 1.10–1.22; *p* < 0.001), and greater intraoperative aspirated hematoma volume (OR 1.05 per 1 mL increase, 95% CI 1.02–1.08; *p* = 0.003) were associated with unfavorable outcome in univariable analysis. In contrast, higher HCR was associated with lower odds of unfavorable outcome (OR 0.88 per 1% increase, 95% CI 0.85–0.92; *p* < 0.001). Barras III–V morphology was strongly associated with unfavorable outcome (OR 16.10, 95% CI 6.97–37.16; *p* < 0.001). Postoperative intracranial rebleeding was also associated with higher odds of unfavorable outcome (OR 9.63, 95% CI 2.13–43.58; *p* = 0.003). Onset-to-surgery time (OR 0.97, 95% CI 0.89–1.07; *p* = 0.583), postoperative intracavitary thrombolysis (OR 0.39, 95% CI 0.07–2.10; *p* = 0.276), and postoperative intracranial infection (OR 2.00, 95% CI 0.35–11.27; *p* = 0.432) were not significantly associated with unfavorable outcome in univariable analysis.

**Table 6:** Multivariable logistic regression analysis of intraoperative aspirated hematoma volume and 30-day unfavorable functional outcome.

Exposure	Crude model		Multivariable-adjusted model 1		Multivariable-adjusted model 2	
	OR (95% CI)	*p*-value	OR (95% CI)	*p*-value	OR (95% CI)	*p*-value
Intraoperative aspirated HV	1.05 (1.02, 1.08)	0.003	0.80 (0.69, 0.92)	0.002	0.78 (0.67, 0.91)	0.002
Intraoperative aspirated HV (quartiles)						
Q1	Ref		Ref		Ref	
Q2	0.51 (0.20, 1.30)	0.161	0.22 (0.05, 1.03)	0.054	0.20 (0.04, 1.10)	0.062
Q3	0.72 (0.29, 1.80)	0.485	0.05 (0.01, 0.37)	0.003	0.03 (0.01, 0.29)	0.002
Q4	2.56 (0.97, 6.75)	0.058	0.02 (0.01, 0.60)	0.025	0.01 (0.01, 0.39)	0.013
*P* for trend		0.049		0.004		0.003

CI, confidence interval; GCS, Glasgow Coma Scale; HV, hematoma volume; INR, international normalized ratio; OR, odds ratio. The outcome was 30-day unfavorable functional outcome, defined as mRS 4–6. For the continuous intraoperative aspirated HV variable, the OR represents the change in odds per 1 mL increase. Crude model: no adjustment. Model 1 adjusted for age, admission GCS category, preoperative hematoma volume, Barras morphology grade, and INR. Model 2 adjusted for Model 1 covariates plus hemoglobin, platelet count, onset-to-surgery time, and postoperative intracavitary thrombolysis. Quartiles of intraoperative aspirated HV were defined according to the sample distribution.

### Multivariable analysis of intraoperative aspirated hematoma volume

The association between intraoperative aspirated hematoma volume and 30-day unfavorable functional outcome is shown in Table 7. In the crude model, greater intraoperative aspirated hematoma volume was associated with higher odds of unfavorable outcome (OR 1.05 per 1 mL increase, 95% CI 1.02–1.08; *p* = 0.003). After adjustment for age, admission GCS category, preoperative hematoma volume, Barras morphology grade, and INR, the direction of association reversed (OR 0.80, 95% CI 0.69–0.92; *p* = 0.002). In the fully adjusted model, which additionally included hemoglobin, platelet count, onset-to-surgery time, and postoperative intracavitary thrombolysis, greater intraoperative aspirated hematoma volume remained associated with lower odds of unfavorable outcome (OR 0.78 per 1 mL increase, 95% CI 0.67–0.91; *p* = 0.002). Quartile-based analysis showed a consistent decreasing trend in unfavorable outcome across higher aspirated-volume quartiles after adjustment. Compared with Q1, the fully adjusted ORs were 0.20 for Q2 (95% CI 0.04–1.10; *p* = 0.062), 0.03 for Q3 (95% CI 0.01–0.29; *p* = 0.002), and 0.01 for Q4 (95% CI 0.01–0.39; *p* = 0.013), with a significant trend across quartiles (*P* for trend = 0.003). Because intraoperative aspirated hematoma volume was highly correlated with preoperative hematoma volume, this analysis was interpreted as supportive rather than as the primary model for surgical clearance efficiency.

**Table 7:** Multivariable logistic regression analysis of hematoma clearance rate and 30-day unfavorable functional outcome.

Exposure	Crude model		Multivariable-adjusted model 1		Multivariable-adjusted model 2	
	OR (95% CI)	*p*-value	OR (95% CI)	*p*-value	OR (95% CI)	*p*-value
HCR	0.88 (0.85, 0.92)	<0.001	0.91 (0.87, 0.96)	<0.001	0.91 (0.86, 0.96)	<0.001
HCR quartiles						
Q1	Ref		Ref		Ref	
Q2	0.37 (0.11, 1.20)	0.097	0.50 (0.10, 2.42)	0.386	0.72 (0.11, 4.02)	0.712
Q3	0.08 (0.02, 0.24)	<0.001	0.12 (0.03, 0.59)	0.009	0.23 (0.04, 1.25)	0.089
Q4	0.02 (0.01, 0.09)	<0.001	0.05 (0.01, 0.34)	0.002	0.03 (0.01, 0.27)	0.002
*P* for trend		<0.001		0.003		0.005

CI, confidence interval; GCS, Glasgow Coma Scale; HCR, hematoma clearance rate; INR, international normalized ratio; OR, odds ratio. The outcome was 30-day unfavorable functional outcome, defined as mRS 4–6. For continuous HCR, ORs are per 1% increase. Crude model: no adjustment. Model 1 adjusted for age, admission GCS category, preoperative hematoma volume, Barras morphology grade, and INR. Model 2 adjusted for Model 1 covariates plus hemoglobin, platelet count, onset-to-surgery time, and postoperative intracavitary thrombolysis. Quartiles were defined according to the sample distribution.

### Multivariable analysis of hematoma clearance rate

The primary HCR analysis is shown in Table 8. In the crude model, higher HCR was associated with lower odds of 30-day unfavorable functional outcome (OR 0.88 per 1% increase, 95% CI 0.85–0.92; *p* < 0.001). This association remained significant after adjustment for age, admission GCS category, preoperative hematoma volume, Barras morphology grade, and INR (OR 0.91, 95% CI 0.87–0.96; *p* < 0.001). In the fully adjusted model, which additionally included hemoglobin, platelet count, onset-to-surgery time, and postoperative intracavitary thrombolysis, higher HCR remained independently associated with lower odds of unfavorable outcome (OR 0.91 per 1% increase, 95% CI 0.86–0.96; *p* < 0.001). In quartile-based analysis, patients in the highest HCR quartile had the lowest odds of unfavorable outcome. Compared with Q1, the fully adjusted ORs were 0.72 for Q2, 0.23 for Q3, and 0.03 for Q4. The trend across HCR quartiles remained significant in the fully adjusted model (*P* for trend = 0.005), supporting a dose–response pattern between higher HCR and lower odds of 30-day unfavorable functional outcome.

**Table 8:** Baseline, perioperative, and outcome characteristics according to the HCR threshold of 60%.

Hematoma clearance rate	HCR < 60%	HCR ≥ 60%	*p*-value
N	76	72	
Age, years	66.08 ± 10.14	59.28 ± 11.84	<0.001
Preoperative hematoma volume, mL	45.82 ± 28.01	31.69 ± 17.84	<0.001
Postoperative residual hematoma volume, mL	24.40 ± 15.05	10.12 ± 6.83	<0.001
Intraoperative aspirated hematoma volume, mL	21.42 ± 14.04	21.57 ± 11.56	0.392
Onset-to-surgery time, hours	6.72 ± 3.33	6.98 ± 3.50	0.643
Sex			0.211
Female	25 (32.89%)	17 (23.61%)	
Male	51 (67.11%)	55 (76.39%)	
Admission GCS score			0.002
3–8 points	52 (68.42%)	29 (40.28%)	
9–12 points	23 (30.26%)	40 (55.56%)	
13–15 points	1 (1.32%)	3 (4.17%)	
Barras morphology grade			<0.001
I-II	15 (19.74%)	47 (65.28%)	
III-V	61 (80.26%)	25 (34.72%)	
Pre HGB, g/L	123.05 ± 23.01	128.50 ± 19.94	0.088
Pre PLT, ×10^9^/L	169.09 ± 68.25	195.04 ± 64.19	0.019
Pre INR	1.22 ± 0.40	1.08 ± 0.13	0.005
Postoperative thrombolysis			0.645
No	3 (3.95%)	4 (5.56%)	
Yes	73 (96.05%)	68 (94.44%)	
Postoperative intracranial rebleeding			0.377
No	65 (85.53%)	65 (90.28%)	
Yes	11 (14.47%)	7 (9.72%)	
Postoperative intracranial infection			0.444
No	72 (94.74%)	70 (97.22%)	
Yes	4 (5.26%)	2 (2.78%)	
30-day functional outcome			<0.001
mRS 0–3	16 (21.05%)	57 (79.17%)	
mRS 4–6	60 (78.95%)	15 (20.83%)	
30-day mortality			0.005
Survival	68 (89.47%)	72 (100.00%)	
Death	8 (10.53%)	0 (0.00%)	

Values are presented as mean ± SD or *n* (%). Continuous variables were compared using the t-test or Mann–Whitney U test, as appropriate. Categorical variables were compared using the chi-square test or Fisher’s exact test when expected cell counts were small. HCR, hematoma clearance rate; GCS, Glasgow Coma Scale; mRS, modified Rankin Scale.

### Characteristics according to the 60% HCR threshold

Patients were further stratified by the approximate ROC-derived HCR threshold of 60%, as shown in Table 9. Compared with patients with HCR ≥ 60%, those with HCR < 60% were older (66.08 ± 10.14 vs. 59.28 ± 11.84 years; *p* < 0.001), had larger preoperative hematoma volumes (45.82 ± 28.01 vs. 31.69 ± 17.84 mL; *p* < 0.001), larger postoperative residual hematoma volumes (24.40 ± 15.05 vs. 10.12 ± 6.83 mL; *p* < 0.001), lower admission GCS scores (GCS 3–8: 68.42% vs. 40.28%; *p* = 0.002), more frequent Barras III–V morphology (80.26% vs. 34.72%; *p* < 0.001), lower platelet counts (169.09 ± 68.25 vs. 195.04 ± 64.19 × 10^9^/L; *p* = 0.019), and higher INR (1.22 ± 0.40 vs. 1.08 ± 0.13; *p* = 0.005). Intraoperative aspirated hematoma volume was similar between groups (21.42 ± 14.04 vs. 21.57 ± 11.56 mL; *p* = 0.392), as was onset-to-surgery time (6.72 ± 3.33 vs. 6.98 ± 3.50 h; *p* = 0.643). Postoperative intracavitary thrombolysis, postoperative intracranial rebleeding, and postoperative intracranial infection did not differ significantly between the HCR groups. Unfavorable 30-day functional outcome occurred in 60 of 76 patients with HCR < 60% and in 15 of 72 patients with HCR ≥ 60% (78.95% vs. 20.83%; *p* < 0.001). Thirty-day mortality occurred only in the HCR < 60% group (10.53% vs. 0%; *p* = 0.005).

**Table 9:** Diagnostic performance of HCR < 60% and postoperative residual hematoma volume >15 mL for predicting 30-day unfavorable functional outcome.

Threshold indicator	Risk-positive definition	TP	FP	FN	TN	Threshold-based AUC	Sensitivity	Specificity	PPV	NPV	Accuracy	PLR	NLR	DOR
HCR threshold	HCR < 60%	60	16	15	57	0.790	0.800	0.781	0.789	0.792	0.791	3.65	0.26	14.25
Residual-volume threshold	Postoperative residual HV > 15 mL	50	10	25	63	0.765	0.667	0.863	0.833	0.716	0.764	4.87	0.39	12.60

The outcome was 30-day unfavorable functional outcome, defined as mRS 4–6. Because HCR ≥60% and postoperative residual hematoma volume ≤15 mL represent favorable surgical-effect thresholds, the corresponding risk-positive definitions for predicting unfavorable outcome were HCR <60% and postoperative residual hematoma volume >15 mL. Threshold-based AUC was calculated as the average of sensitivity and specificity. AUC, area under the curve; DOR, diagnostic odds ratio; HCR, hematoma clearance rate; HV, hematoma volume; FN, false negative; FP, false positive; NLR, negative likelihood ratio; NPV, negative predictive value; PLR, positive likelihood ratio; PPV, positive predictive value; TN, true negative; TP, true positive.

### Smooth associations of HCR and intraoperative aspirated hematoma volume with outcome

**Figure 3:**
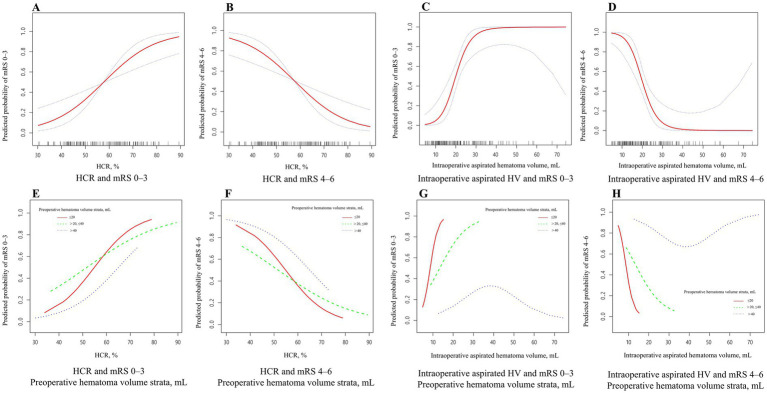
Smooth associations of hematoma clearance rate and intraoperative aspirated hematoma volume with 30-day functional outcome after sMIS. **(A,B)** show generalized additive logistic models for the predicted probability of 30-day mRS 0–3 and mRS 4–6 across hematoma clearance rate (HCR, %). **(C,D)** show the corresponding predicted probabilities across intraoperative aspirated hematoma volume. **(E,F)** show HCR–outcome curves stratified by preoperative hematoma volume strata (≤20 mL, >20–40 mL, and >40 mL), and **(G,H)** show intraoperative aspirated hematoma volume–outcome curves within the same strata. Curves indicate fitted predicted probabilities, and dashed lines indicate 95% confidence intervals where shown. Tick marks on the x-axes indicate the distribution of observations. Models were adjusted for age, admission GCS category, Barras morphology grade, INR, hemoglobin, platelet count, onset-to-surgery time, and postoperative intracavitary thrombolysis. Preoperative hematoma volume was additionally included as a covariate except when used for stratification. GCS, Glasgow Coma Scale; HCR, hematoma clearance rate; HV, hematoma volume; INR, international normalized ratio; mRS, modified Rankin Scale; sMIS, stereotactic minimally invasive surgery.

Generalized additive logistic models were used to visualize the associations of HCR and intraoperative aspirated hematoma volume with 30-day outcome, as shown in Figure 3. In the overall cohort, increasing HCR was associated with a higher predicted probability of favorable outcome (mRS 0–3) and a lower predicted probability of unfavorable outcome (mRS 4–6). This pattern was directionally consistent across preoperative hematoma volume strata, although the slope of the HCR–outcome association varied by baseline hematoma volume. Intraoperative aspirated hematoma volume also showed nonlinear associations with 30-day outcome. However, compared with HCR, the association of absolute aspirated volume with outcome varied more across preoperative hematoma volume strata. These curves suggest that HCR, which incorporates both baseline hematoma size and postoperative residual clot burden, may provide a more stable measure of relative surgical clearance efficiency than absolute aspirated volume alone.

### ROC analysis and threshold-based diagnostic performance

**Figure 4:**
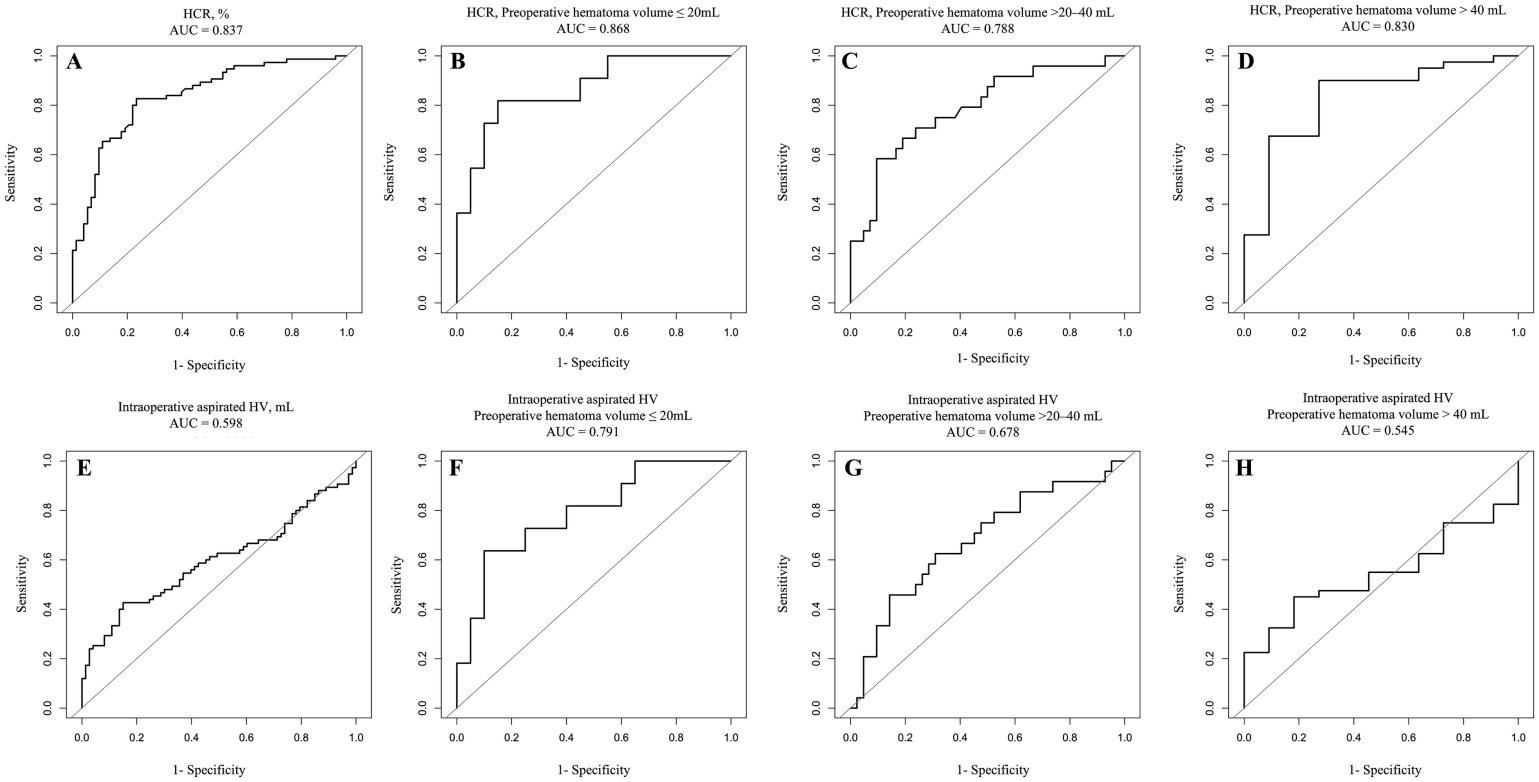
Receiver-operating characteristic curves for predicting 30-day unfavorable functional outcome after sMIS. **(A–D)** show ROC curves for hematoma clearance rate (HCR, %) in the overall cohort and across preoperative hematoma volume strata: overall cohort, ≤20 mL, >20–40 mL, and >40 mL. **(E–H)** show ROC curves for intraoperative aspirated hematoma volume in the overall cohort and across the same preoperative hematoma volume strata. The outcome was 30-day unfavorable functional outcome, defined as mRS 4–6. The diagonal gray line denotes non-discrimination. Optimal cut-offs were determined using the Youden index. AUC, area under the curve; HCR, hematoma clearance rate; HV, hematoma volume; mRS, modified Rankin Scale; ROC, receiver-operating characteristic; sMIS, stereotactic minimally invasive surgery.

ROC analyses showed that HCR had good discrimination for 30-day unfavorable functional outcome, with an overall AUC of 0.837 and an approximate optimal threshold near 60%, as shown in Figure 4. Stratified ROC analyses showed that HCR retained good discrimination across preoperative hematoma volume strata, with AUCs of 0.868 for preoperative hematoma volume ≤20 mL, 0.788 for >20–40 mL, and 0.830 for >40 mL. In contrast, intraoperative aspirated hematoma volume showed more modest discrimination overall (AUC 0.598) and variable performance across strata, with AUCs of 0.791 for ≤20 mL, 0.678 for >20–40 mL, and 0.545 for >40 mL. We further compared the threshold-based diagnostic performance of HCR < 60% and postoperative residual hematoma volume >15 mL for identifying 30-day unfavorable functional outcome, as shown in Table 10. HCR < 60% showed higher sensitivity, negative predictive value, accuracy, and threshold-based AUC than postoperative residual hematoma volume >15 mL, whereas the residual-volume threshold showed higher specificity and positive predictive value. Specifically, HCR < 60% yielded a sensitivity of 0.800, specificity of 0.781, positive predictive value of 0.789, negative predictive value of 0.792, accuracy of 0.791, and diagnostic odds ratio of 14.25. Postoperative residual hematoma volume >15 mL yielded a sensitivity of 0.667, specificity of 0.863, positive predictive value of 0.833, negative predictive value of 0.716, accuracy of 0.764, and diagnostic odds ratio of 12.60.

**Table 10:** Multicollinearity diagnostics for the fully adjusted multivariable models.

Variable	VIF in intraoperative aspirated HV model	VIF in HCR model
Intraoperative aspirated HV, mL	7.88	—
HCR, %	—	1.50
Age, years	1.25	1.25
Admission GCS 9–12 vs. 3–8	1.25	1.28
Admission GCS 13–15 vs. 3–8	1.08	1.08
Preoperative HV, mL	9.82	1.53
Barras III–V vs. I–II	1.62	1.76
INR	1.19	1.16
Hemoglobin, g/L	1.23	1.22
Platelet count, ×10^9^/L	1.19	1.19
Onset-to-surgery time, h	1.07	1.06
Postoperative intracavitary thrombolysis, yes vs. no	1.12	1.12

VIF values were calculated for the fully adjusted multivariable logistic regression models. Admission GCS category was dummy-coded with GCS 3–8 as the reference group. Barras morphology grade was coded as III–V versus I–II. VIF <5 was considered to indicate no substantial multicollinearity. HCR, hematoma clearance rate; HV, hematoma volume; INR, international normalized ratio; VIF, variance inflation factor.

### Multicollinearity, interaction, sensitivity, and reliability analyses

Multicollinearity diagnostics are shown in Table 11. No substantial collinearity was observed in the primary HCR model, with all VIF values below 2.0. The VIF for HCR was 1.50, and the VIFs for the covariates in the HCR model ranged from 1.06 to 1.76. In the intraoperative aspirated hematoma volume model, VIF values were elevated for intraoperative aspirated HV and preoperative HV, with VIFs of 7.88 and 9.82, respectively. This was consistent with the expected correlation between baseline hematoma burden and aspirated volume. Therefore, the aspirated-volume analysis was interpreted as supportive, whereas the HCR model was regarded as the primary model for evaluating surgical clearance efficiency. Interaction analyses are shown in Table 12. No statistically significant interaction was observed between HCR and preoperative hematoma volume strata. The interaction ORs were 0.99 for >20–40 mL versus ≤20 mL (95% CI 0.85–1.16; *p* = 0.925) and 0.97 for >40 mL versus ≤20 mL (95% CI 0.81–1.16; *p* = 0.727), with an overall P for interaction of 0.927. Similarly, the HCR × Barras morphology interaction was not statistically significant (OR 1.00, 95% CI 0.89–1.12; *p* = 0.999). These findings suggest that the association between HCR and 30-day unfavorable outcome was broadly consistent across baseline hematoma-volume strata and morphology groups.

**Table 11:** Interaction analyses for HCR and 30-day unfavorable functional outcome.

Interaction term	Reference group	Interaction comparison	Interaction OR	95% CI	P for interaction
HCR × preoperative hematoma volume strata	≤20 mL	>20–40 mL vs. ≤ 20 mL	0.99	0.85–1.16	0.925
HCR × preoperative hematoma volume strata	≤20 mL	>40 mL vs. ≤ 20 mL	0.97	0.81–1.16	0.727
Overall HCR × preoperative hematoma volume strata	—	3 strata overall interaction	—	—	0.927
HCR × Barras morphology grade	Barras I–II	Barras III–V vs. I–II	1.00	0.89–1.12	0.999

The outcome was 30-day unfavorable functional outcome, defined as mRS 4–6. HCR was modeled as a continuous variable per 1% increase. For the HCR × preoperative hematoma volume interaction model, preoperative hematoma volume was entered as strata (≤20 mL, >20–40 mL, and >40 mL), and the model was adjusted for age, admission GCS category, Barras morphology grade, INR, hemoglobin, platelet count, onset-to-surgery time, and postoperative intracavitary thrombolysis. For the HCR × Barras morphology interaction model, the model was adjusted for age, admission GCS category, preoperative hematoma volume, INR, hemoglobin, platelet count, onset-to-surgery time, and postoperative intracavitary thrombolysis. CI, confidence interval; HCR, hematoma clearance rate; INR, international normalized ratio; mRS, modified Rankin Scale; OR, odds ratio.

**Table 12:** Sensitivity analysis additionally adjusting for postoperative intracranial rebleeding and intracranial infection.

Exposure	Crude model		Multivariable-adjusted model 1		Sensitivity model	
	OR (95% CI)	*p*-value	OR (95% CI)	*p*-value	OR (95% CI)	*p*-value
HCR	0.88 (0.85, 0.92)	<0.001	0.91 (0.87, 0.96)	<0.001	0.89 (0.84–0.95)	<0.001
HCR quartiles						
Q1	Ref		Ref		Ref	
Q2	0.37 (0.11, 1.20)	0.097	0.50 (0.10, 2.42)	0.386	0.58 (0.09, 3.65)	0.563
Q3	0.08 (0.02, 0.24)	<0.001	0.12 (0.03, 0.59)	0.009	0.17 (0.03, 1.05)	0.056
Q4	0.02 (0.01, 0.09)	<0.001	0.05 (0.01, 0.34)	0.002	0.02 (0.01, 0.19)	<0.001
*P* for trend		<0.001		0.003		<0.001

The outcome was 30-day unfavorable functional outcome, defined as mRS 4–6. HCR was modeled per 1% increase and by quartiles. Adjusted Model 1 included age, admission GCS category, preoperative hematoma volume, Barras morphology grade, and INR. The sensitivity model further adjusted for hemoglobin, platelet count, onset-to-surgery time, postoperative intracavitary thrombolysis, postoperative intracranial rebleeding, and postoperative intracranial infection. HCR, hematoma clearance rate; INR, international normalized ratio; OR, odds ratio. The sensitivity model was based on the fully adjusted HCR model and additionally included postoperative intracranial rebleeding and postoperative intracranial infection.

Sensitivity analysis additionally adjusting for postoperative intracranial rebleeding and postoperative intracranial infection is shown in Table 13. After this additional adjustment, higher HCR remained significantly associated with lower odds of 30-day unfavorable functional outcome (OR 0.89 per 1% increase, 95% CI 0.84–0.95; *p* < 0.001). Quartile-based analysis remained directionally consistent. Compared with Q1, the sensitivity-model ORs were 0.58 for Q2 (95% CI 0.09–3.65; *p* = 0.563), 0.17 for Q3 (95% CI 0.03–1.05; *p* = 0.056), and 0.02 for Q4 (95% CI 0.01–0.19; *p* < 0.001), with a significant trend across quartiles (*P* for trend<0.001). Inter-rater reliability results are shown in Table 14. Inter-rater reliability was excellent for both Barras morphology grading and hematoma volumetry. The weighted Cohen’s *κ* for Barras grades I–V was 0.995 (95% CI 0.988–1.000), and the Cohen’s κ for the dichotomized Barras morphology group was 0.972 (95% CI 0.929–1.000). The ICCs for preoperative and postoperative residual hematoma volume measurements were 0.999 (95% CI 0.999–0.999) and 0.999 (95% CI 0.999–1.000), respectively.

**Table 13:** Inter-rater reliability of Barras grading and hematoma volume measurements.

Measurement	Sample size	Reliability metric	Estimate	95% CI
Barras grade I–V	148	Weighted Cohen’s κ	0.995	0.988–1.000
Barras morphology group, I–II vs. III–V	148	Cohen’s κ	0.972	0.929–1.000
Preoperative hematoma volume	148	ICC	0.999	0.999–0.999
Postoperative residual hematoma volume	148	ICC	0.999	0.999–1.000

Barras grade I–V was assessed using weighted Cohen’s κ. The dichotomized Barras morphology group was defined as grades I–II versus III–V and assessed using Cohen’s κ. Intraclass correlation coefficients were calculated using a two-way random-effects model for absolute agreement. CI, confidence interval; ICC, intraclass correlation coefficient.

**Table 14:** Comparison of key baseline characteristics between included and excluded patients.

Variable	Included patients (*n* = 148)	Excluded patients (*n* = 21)	*p*-value
Age, years	62.77 ± 11.48	63.5 ± 13.2	0.812
Sex, male, *n* (%)	106 (71.6%)	14 (66.7%)	0.640
Smoking history, *n* (%)	37 (25.0%)	8 (38.1%)	0.204
Drinking history, *n* (%)	28 (18.9%)	5 (23.8%)	0.565
Hypertension, *n* (%)	122 (82.4%)	16 (76.2%)	0.547
History of ICH, *n* (%)	19 (12.8%)	3 (14.3%)	0.740
History of cerebral infarction, *n* (%)	26 (17.6%)	2 (9.5%)	0.533
Admission GCS, median (IQR)	8 (5–11)	7 (5–9)	Not tested
Preoperative hematoma volume, mL	38.95 ± 24.58	46.8 ± 32.4	0.296
Barras grade, median (IQR)	3 (2–5)	4 (3–5)	Not tested
Admission SBP, mmHg	156.4 ± 34.0	163.0 ± 35.5	0.432
Admission DBP, mmHg	89.3 ± 18.5	92.0 ± 19.1	0.544
Preoperative INR	1.15 ± 0.31	1.19 ± 0.36	0.636
Onset-to-surgery time, h, median (IQR)	6.6 (3.8–9.5)	5.4 (3.2–9.5)	Not tested

Values are presented as mean ± SD, median (IQR), or *n* (%). Continuous variables reported as mean ± SD were compared using Welch’s *t*-test. Categorical variables were compared using the chi-square test or Fisher’s exact test, as appropriate. *p*-values were not calculated for variables available only as median (IQR) in the excluded cohort because individual-level data were not available. DBP, diastolic blood pressure; GCS, Glasgow Coma Scale; ICH, intracerebral hemorrhage; INR, international normalized ratio; IQR, interquartile range; SBP, systolic blood pressure.

## Discussion

In this single-center retrospective cohort of patients with spontaneous supratentorial ICH treated with sMIS, we found that HCR was independently associated with 30-day functional outcome after adjustment for baseline clinical severity, preoperative hematoma volume, hematoma morphology, coagulation status, onset-to-surgery time, and postoperative intracavitary thrombolysis. In the fully adjusted model, each 1% increase in HCR was associated with lower odds of 30-day unfavorable functional outcome, and patients in the highest HCR quartile had the lowest odds of mRS 4–6. This association remained directionally consistent in sensitivity analyses additionally adjusting for postoperative intracranial rebleeding and intracranial infection. Together, these findings support HCR as a postoperative prognostic benchmark after sMIS. However, because this study was observational, HCR should be interpreted as an associative marker rather than direct evidence that achieving a specific clearance threshold causally improves outcome. A central finding of this study was that an HCR threshold near 60% showed good discrimination for 30-day unfavorable outcome. Patients with HCR < 60% had worse baseline and postoperative profiles than those with HCR ≥ 60%, including older age, larger preoperative hematoma volume, larger postoperative residual hematoma volume, lower admission GCS, more frequent Barras III–V morphology, lower platelet count, and higher INR. They also had a substantially higher proportion of 30-day unfavorable functional outcome and higher 30-day mortality. These findings suggest that the 60% threshold identified in our cohort separated clinically distinct risk groups. Nevertheless, this threshold should not be interpreted as a rigid procedural target. Patients with lower HCR also had worse baseline features, suggesting that lower clearance may partly reflect more severe or technically challenging hematomas rather than a modifiable surgical factor alone.

The approximately 60% threshold identified in our cohort should be interpreted in the context of previous minimally invasive surgical evidence. In MISTIE III, minimally invasive surgery with thrombolysis did not significantly improve the primary functional endpoint in the overall trial population, but analyses from the trial emphasized the importance of effective clot reduction and small residual hematoma volume, particularly the residual-volume benchmark of approximately 15 mL (17). Differences between our 60% HCR threshold and previously reported higher evacuation thresholds may reflect differences in patient selection, surgical technique, hematoma location, baseline hematoma-volume distribution, morphology, thrombolysis protocols, timing of postoperative imaging, and outcome time points. The ENRICH trial further supports contemporary interest in early minimally invasive hematoma removal with careful patient selection and standardized procedural strategies (18). Therefore, the 60% value should be regarded as a cohort-derived prognostic benchmark that requires prospective external validation, rather than as evidence that 60% clearance is universally sufficient for all patients.

Our comparison between HCR and the residual-volume threshold of 15 mL provides further context. Residual hematoma volume reflects the absolute postoperative clot burden, whereas HCR incorporates baseline hematoma size and therefore reflects relative evacuation efficiency. In our threshold-based analysis, HCR < 60% showed higher sensitivity, NPV, accuracy, and threshold-based AUC for identifying 30-day unfavorable outcome, whereas postoperative residual hematoma volume >15 mL showed higher specificity and PPV. This suggests that the two indicators capture related but distinct aspects of surgical efficacy. HCR may be useful for risk stratification across a broad range of baseline hematoma volumes, while residual volume may be particularly useful for identifying patients with a high absolute postoperative clot burden. Rather than competing with each other, HCR and residual-volume thresholds may be complementary metrics for postoperative assessment after sMIS, consistent with the emphasis on effective clot reduction and low residual hematoma burden in MISTIE III (17).

The role of baseline hematoma volume deserves particular attention. In the generalized additive models and ROC analyses, HCR retained good discriminative performance across preoperative hematoma-volume strata, although the shape and slope of the association varied by baseline volume. Interaction analysis did not show significant effect modification by preoperative hematoma-volume strata, suggesting that the association between HCR and 30-day outcome was broadly consistent across the volume groups examined. Clinically, however, baseline volume remains important because it influences both the feasibility of evacuation and the residual clot burden after surgery. Larger hematomas may allow greater absolute aspiration volume but still leave substantial residual clot, whereas smaller hematomas may achieve low residual volume with more modest absolute removal. This may explain why intraoperative aspirated hematoma volume showed elevated collinearity with preoperative hematoma volume and was interpreted as a supportive rather than primary measure of surgical clearance efficiency. Prior analyses of surgical decision-making in ICH trials also support the concept that surgical benefit is highly dependent on patient selection, hematoma characteristics, and procedural context (19).

Hematoma morphology was another important factor. Barras III–V morphology was strongly associated with unfavorable outcome in baseline and univariable analyses, indicating that irregular hematoma shape may reflect a higher-risk phenotype. Prior studies have shown that hematoma shape, density, and other CT-derived characteristics are associated with hematoma expansion and clinical prognosis in ICH (20–22). Irregular or multilobulated hematomas may be more difficult to evacuate completely, may have more complex clot geometry, and may carry higher risk of residual clot or rebleeding. However, the HCR × Barras morphology interaction was not statistically significant, suggesting no clear evidence that morphology modified the association between HCR and 30-day outcome. Thus, Barras morphology should be viewed as an important baseline risk and procedural complexity marker, but not necessarily as evidence that HCR loses prognostic relevance in irregular hematomas. Reliable imaging measurement is essential when HCR is used as a quantitative postoperative marker. In this study, hematoma volume was measured using 3D Slicer-based segmentation rather than simple linear diameter estimation. Computerized hematoma volume estimation has been shown to provide more precise quantification than conventional ABC-based methods in ICH research (23). In our cohort, inter-rater reliability was excellent for both Barras grading and preoperative/postoperative hematoma volume measurement, supporting the reproducibility of the imaging-derived variables used in the analysis.

Onset-to-surgery time did not differ significantly between favorable and unfavorable outcome groups and was not significant in univariable analysis, but it was included in the fully adjusted models because of its clinical relevance. Previous work has suggested that surgical timing may influence perihematomal tissue injury and biological responses after hypertensive ICH (24). Postoperative intracavitary thrombolysis was used in most patients and did not differ significantly between outcome groups, but it was also included in the fully adjusted models because thrombolysis may influence residual clot burden and HCR. Postoperative intracranial rebleeding was more frequent in patients with unfavorable outcome and was strongly associated with poor outcome in univariable analysis. Importantly, the HCR–outcome association remained significant after additional adjustment for postoperative intracranial rebleeding and intracranial infection, suggesting that the prognostic association of HCR was not solely explained by these major postoperative complications. Even so, postoperative rebleeding remains clinically important and should be carefully considered when interpreting early postoperative outcomes after sMIS.

From a clinical perspective, the present findings support the use of HCR as a quantitative postoperative assessment marker after sMIS. HCR is attractive because it is simple to calculate from preoperative and postoperative CT volumetry and reflects the relative degree of clot removal. In settings where 3D volumetry is available, HCR may help standardize reporting of surgical clearance, support postoperative risk stratification, and facilitate comparison across studies. Current guideline summaries continue to emphasize individualized management, careful patient selection, and multidisciplinary decision-making in spontaneous ICH (25). Therefore, the findings of this study should not be interpreted as proof that aggressive pursuit of a specific HCR threshold will improve outcomes. Attempts to achieve higher clearance must be balanced against procedural safety, especially in elderly patients, patients with low admission GCS, irregular Barras III–V morphology, coagulopathy, or large hematomas. Prospective studies are needed to determine whether HCR-guided surgical strategies can improve patient-centered outcomes beyond standard clinical decision-making.

## Limitation

This study has several limitations. First, it was a single-center retrospective cohort with a modest sample size, which limits generalizability and leaves the possibility of residual confounding. Although we adjusted for major clinical, imaging, laboratory, and perioperative variables, unmeasured factors such as rehabilitation intensity, postoperative blood-pressure variability, neurocritical care differences, edema progression, and detailed thrombolysis dosing may still have influenced 30-day outcomes. Second, the primary endpoint was 30-day mRS, and longer-term functional recovery was not assessed. Third, although we clarified the reasons for exclusion and compared key baseline characteristics between included and excluded patients, selection bias cannot be fully excluded because some excluded patients lacked complete imaging or follow-up data. Fourth, the HCR threshold was derived using the Youden index in this cohort, and threshold estimates may vary across populations, imaging timing, surgical techniques, and outcome definitions. Therefore, the 60% threshold requires prospective validation before it can be used as a prescriptive treatment target. Fifth, the cohort was drawn from a Chinese single-center population, which may limit external validity to other health-care systems, populations, or sMIS techniques. Finally, although inter-rater reliability for Barras grading and hematoma volumetry was excellent, imaging-based measurements may still be influenced by CT acquisition parameters, segmentation workflow, and postoperative artifact.

## Conclusion

In patients with supratentorial ICH undergoing sMIS, higher postoperative hematoma clearance rate was independently associated with lower odds of 30-day unfavorable functional outcome. An HCR threshold near 60% may serve as a pragmatic postoperative prognostic benchmark and may complement absolute residual-volume thresholds such as 15 mL. Because this was an observational study, this threshold should be interpreted as a risk-stratification marker rather than as a prescriptive procedural target. Prospective multicenter studies are needed to validate the prognostic value of HCR and to determine whether HCR-guided surgical strategies can improve patient-centered outcomes.

## Data Availability

The original contributions presented in the study are included in the article/supplementary material, further inquiries can be directed to the corresponding author.
